# Evidence for Habitual and Goal-Directed Behavior Following Devaluation of Cocaine: A Multifaceted Interpretation of Relapse

**DOI:** 10.1371/journal.pone.0007170

**Published:** 2009-09-25

**Authors:** David H. Root, Anthony T. Fabbricatore, David J. Barker, Sisi Ma, Anthony P. Pawlak, Mark O. West

**Affiliations:** Department of Psychology, Rutgers University, New Brunswick, New Jersey, United States of America; Freie Universitaet Berlin, Germany

## Abstract

**Background:**

Cocaine addiction is characterized as a chronically relapsing disorder. It is believed that cues present during self-administration become learned and increase the probability that relapse will occur when they are confronted during abstinence. However, the way in which relapse-inducing cues are interpreted by the user has remained elusive. Recent theories of addiction posit that relapse-inducing cues cause relapse habitually or automatically, bypassing processing information related to the consequences of relapse. Alternatively, other theories hypothesize that relapse-inducing cues produce an expectation of the drug's consequences, designated as goal-directed relapse. Discrete discriminative stimuli signaling the availability of cocaine produce robust cue-induced responding after thirty days of abstinence. However, it is not known whether cue-induced responding is a goal-directed action or habit.

**Methodology/Principal Findings:**

We tested whether cue-induced responding is a goal-directed action or habit by explicitly pairing or unpairing cocaine with LiCl-induced sickness (n = 7/group), thereby decreasing or not altering the value of cocaine, respectively. Following thirty days of abstinence, no difference in responding between groups was found when animals were reintroduced to the self-administration environment alone, indicating habitual behavior. However, upon discriminative stimulus presentations, cocaine-sickness paired animals exhibited decreased cue-induced responding relative to unpaired controls, indicating goal-directed behavior. In spite of the difference between groups revealed during abstinent testing, no differences were found between groups when animals were under the influence of cocaine.

**Conclusions/Significance:**

Unexpectedly, both habitual and goal-directed responding occurred during abstinent testing. Furthermore, habitual or goal-directed responding may have been induced by cues that differed in their correlation with the cocaine infusion. Non-discriminative stimulus cues were weak correlates of the infusion, which failed to evoke a representation of the value of cocaine and led to habitual behavior. However, the discriminative stimulus–nearly perfectly correlated with the infusion–likely evoked a representation of the value of the infusion and led to goal-directed behavior. These data indicate that abstinent cue-induced responding is multifaceted, dynamically engendering habitual or goal-directed behavior. Moreover, since goal-directed behavior terminated habitual behavior during testing, therapeutic approaches aimed at reducing the perceived value of cocaine in addicted individuals may reduce the capacity of cues to induce relapse.

## Introduction

One of the most insidious characteristics of cocaine addiction is its chronic relapsing nature [1]. It is believed that various types of cues (paraphernalia, drug-associated odors and sounds, availability of cocaine, drug-use partners, etc) present during cocaine self-administration become learned and increase the probability that relapse will occur when an abstinent user is confronted with these cues [1], [2]. However, the behavioral mechanism that underlies cue-induced relapse is poorly understood. Recent theories of the neural basis of addiction posit that habit formation is a necessary contributor [2]–[6]. Habits have been defined as automatic behaviors that are insensitive to manipulations of their consequences [7]. Given that cocaine addiction is associated with a high risk of relapse despite negative consequences of returning to drug use such as sickness, depression, or loss of employment [1], it is reasonable to hypothesize that cue-elicited relapse is a habitual behavior. An alternate hypothesis [8] suggests that cues elicit an expectation of drug which drives drug-seeking (a goal-directed action). Distinguishing between goal-directed and habitual responding can be accomplished by manipulation of the response outcome [7], cocaine. In animal research, either of two methods, satiation of the reward or pairing the reward with an unpleasant outcome such as sickness, reduce the reward's “value” to the animal [7]. If either of these methods reduces the number of responses emitted in order to earn the reward in the devalued relative to the normal valued reward group, then the behavior is interpreted as a goal-directed action. In contrast, if there is no difference between devalued and valued groups, the behavior is deemed a stimulus-bound habit.

While experimenter-administered psychostimulants such as cocaine have been shown to enhance the formation of habitual responding for food [9]–[11], few reports have investigated whether cocaine self-administration behavior is controlled by habit. Dickinson and colleagues have demonstrated that oral sweetened cocaine-seeking behavior can become habitual [12], as tested by pairing the oral solution with LiCl-induced sickness. However, this report utilized an oral sweetened-cocaine solution as the reward, not the long utilized intravenous cocaine self-administration paradigm [13]. Furthermore, human cocaine self-administration studies have suggested that the intravenous route of self-administration has markedly greater potential for abuse than oral self-administration [14].

A recent report by Norman and Tsibulsky [15] found that intravenous cocaine self-administration behavior was completely blocked by satiety, and thus a goal-directed action rather than habitual. They reported that responding on the manipulandum that produced drug delivery, i.e., cocaine-seeking behavior, while under the influence of the drug was binary. When the animal was under the influence of cocaine, but not satiated, it exhibited ‘compulsive’ responding. Yet, when cumulative cocaine infusions reached the animal's ‘satiety’ level, the animal did not respond. One interpretation of those findings, although not new, is that drug level determines the rate of responding [13], [16].

Thus, while drug binge behavior may be goal-directed, it is unknown whether cue-elicited relapse, which is under abstinent conditions, is a goal-directed action or a stimulus-bound habit. While the link between craving and relapse is not completely understood [17], cues that signal the availability of cocaine (and other drugs) are potent producers of craving for the drug in cue-reactive individuals [18]–[21]. We have previously shown that discrete discriminative stimuli (S^D^) that signal the availability of cocaine stimulate responding in rats following thirty days of forced abstinence [22]. In the present experiment, we tested whether cue-elicited relapse is a goal-directed action or a stimulus-bound habit by manipulating the “value” of cocaine via explicit pairings of cocaine with LiCl-induced sickness. Control animals received LiCl treatments that were not paired with cocaine. If cue-induced responding is goal-directed, response rates of paired animals should be lower than unpaired animals. In contrast, if cue-induced responding is a habit, response rates between paired and unpaired animals should not differ. Given that cocaine self-administration behavior is goal-directed [15], we hypothesized that cue-induced responding following thirty days of abstinence is also goal-directed.

## Results

**Figure 1:**
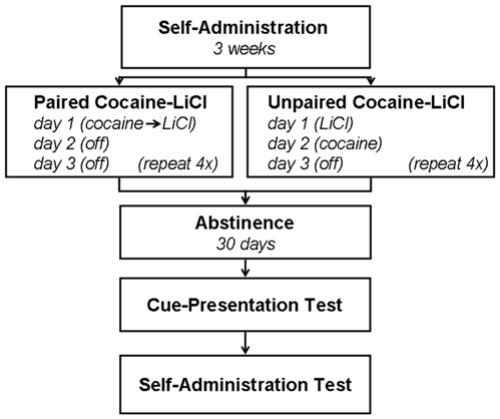
Experimental timeline. Following three weeks of cocaine self-administration, subjects were administered cocaine or LiCl on the same day (paired) or on separate days (unpaired). After thirty days of forced abstinence, animals were returned to the self-administration chambers for cue-presentation and self-administration tests.

The overall experimental schematic is presented in Figure 1( see Methods). Consistent with various paradigms [15], [16], [22], [23], all animals learned to self-administer cocaine, increasing daily response rates and drug consumed over weeks of extended training. Over three weeks of self-administration training and prior to LiCl treatments, rats in both groups increased self-administered cocaine (F(2, 24) = 13.49, P<.0001) to a daily consumption level of 28.978±0.493 mg/kg/day (LiCl paired: 28.66±0.44; LiCl unpaired: 29.297±0.907) and increased the number of responses/min/day (F(2, 24) = 8.19, p<.05) to an average of 7.049±2.814 (LiCl paired: 10.011±5.539; LiCl unpaired: 4.047±0.840) in the third week. Although no statistical difference was found between groups, one outlier animal in the paired group (average responses/min/day was 41.632) inflated the average daily responses/min in the paired group (average responses/min/day without this animal: 4.740±1.869). Furthermore, there was no difference in time spent self-administering cocaine in either group (t(12) = 1.269, p>.05), averaging 5.790±0.041 hours/day for both groups in the third week of training (paired: 5.739±0.045; unpaired: 5.840±0.066). Thus, animals increased consumption of cocaine as well as the number of responses emitted. The ratio of total responses over total earned rewards significantly increased over weeks of self-administration (F(2,24) = 7.10, p<.01) to an average level of 33.043±12.869 in the third week (paired: 46.850±25.310; paired without outlier rat: 22.824±9.415; unpaired: 19.235±3.676). Such increasing response∶reward ratios are consistent with those hypothesized to be critical in habit formation [7], [24]. There was no group difference (p>.05) or week × group interaction (p>.05) for any measure. On the last day of training the unpaired group emitted 4.748±1.117 responses/min, self-administering 30.193±0.551 mg/kg of cocaine, and the paired group emitted 14.74±9.631 (5.202±1.573 without outlier animal) responses/min, self-administering 29.726±0.493 mg/kg of cocaine.

Although all rats significantly decreased S^D^ reaction times (including both hits and misses) over weeks of training to 0.315±0.035 min/day in the third week of training (paired: 0.326±0.051; unpaired: 0.304±0.052), F(2,24) = 22.51, p<.0001, animals did not discriminate responding between S^D^ presence or absence, consistent with our previous study using the same schedule of reinforcement [22]. There was no significant group effect (p>.05) or week × group interaction (p>.05). On the last day of training, mean reaction time to the S^D^ for the paired group was 0.243±0.042 min and the unpaired group was 0.296±0.047 min.

Based on week three training data, we estimated the correlations of the S^D^, lever, and operant chamber with the self-administered cocaine infusion. Rats responded on average 75.521 times out of an average 77.934 S^D^ presentations, giving the S^D^ the strongest correlation with the cocaine infusion at 0.969. In contrast, based on response∶reward ratio's of 33.043 responses for a single infusion, the lever press had a 0.030 correlation with cocaine infusions. Furthermore, based on the maximum (80) number of 3.755-sec infusions of cocaine per day divided by 24 hours, the self-administration chamber had a 0.003 correlation with cocaine infusions. Thus, the S^D^, but not the lever press or self-administration chamber, was highly correlated with the outcome, or infusion of cocaine.

During the devaluation phase of the experiment, there was no difference in total LiCl administered between groups (t(12) = 0.096, p>0.05), averaging 898.714±63.778 mg/kg/rat (paired: 905.143±109.751; unpaired: 892.286±74.613). Animals rarely exhibited “lying on belly”, “sickness” behavior or diarrhea, similar to a previous report using intravenous LiCl administration [25].

**Figure 2:**
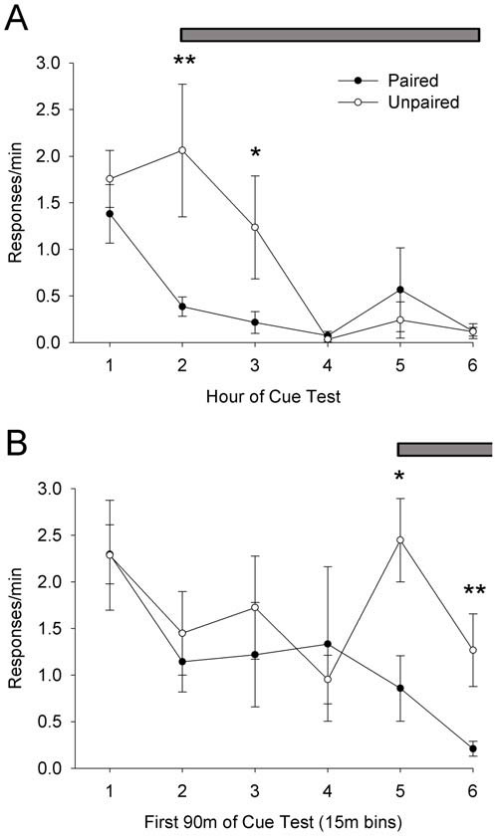
Cue-presentation test. A. Responses/minute of paired and unpaired animals over the six hour cue-presentation test. B. Responses/minute of paired and unpaired animals over the first ninety minutes of the cue-presentation test. Horizontal bars indicate period of variable 3–6 min interval S^D^ tone presentations, variably presented every 3–6 min. N = 7 for both paired and unpaired groups.

With respect to the cue-presentation phase of the experiment, a mixed ANOVA yielded a significant main effect of hour, suggesting that over six hours of testing under abstinence (extinction) conditions, both paired and unpaired animals decreased response rates, F(5, 60) = 10.75, p<. 0001, with no overall group difference, F(1, 60) = 0.41, p>.05. However, this analysis also yielded a significant hour × group interaction, F(5, 60) = 3.12, p<.05, indicating that groups differed in their response rates depending on the hour of the cue-test (Figure 2A). Post-hoc simple comparisons revealed that response rates during the hour in which S^D^ cues were not present (hour 1) did not differ between groups, F(1, 60) = 0.60, p>.05. In contrast, during the first two hours in which the cocaine-associated S^D^ was present (hours 2 and 3), the unpaired group exhibited significantly higher response rates than the paired group (second hour: F(1, 60) = 12.65, p<.001; third hour: F(1, 60) = 5.29, p<.05). No differences between groups were found for the remainder of the hours.

These results suggest that cue-induced responding under abstinent conditions is decreased by prior devaluation of cocaine. However, it is possible that animals in the paired group extinguished to the return of the operant environment more quickly than animals in the unpaired group. To test this possibility, we analyzed the first 90 minutes of the cue-presentation session in fifteen minute bins. While the ANOVA yielded a significant main effect of bin, F(5, 60) = 6.06, p<.001 and no main effect of group, F(1, 60) = 2.94, p>.05, a significant bin × group interaction was revealed, F(5, 60) = 3.30, p<.01. Post-hoc simple comparisons revealed that response rates prior to S^D^ presentations did not differ between groups (Figure 2B). However, during the first fifteen minutes of S^D^ presentations (bin 5), a significant difference between paired and unpaired animals emerged, F(1, 60) = 4.70, p<.05, and continued throughout the first thirty minutes of S^D^ presentations (bin 6), F(1, 60) = 11.76, p<.01. Thus, prior devaluation of cocaine produced significant differences in response rates between paired and unpaired groups selectively during S^D^-exposure. Since groups contrasted in response rates only following the onset of S^D^ presentations, it is likely that the LiCl-paired devaluation of cocaine reduced S^D^-induced responding rather than an acceleration of extinction responding upon returning to the operant environment. That is, the SD failed to excite responding in the paired group.

**Figure 3:**
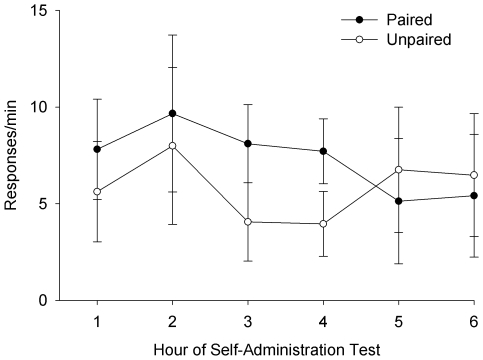
Post-devaluation cocaine self-administration test. Responses/minute of paired and unpaired animals over the six hour self-administration test. N = 7 for both paired and unpaired groups.

One day following the cue-presentation test, animals were given access to cocaine using the same protocol as used during the self-administration phase of the experiment. No overall differences in drug intake (mg/kg; t(12) = 0.32, p>.05), response rates (t(12) = −0.70, p>.05), or S^D^ reaction times (hits and misses; t(12) = 0.39, p>.05) were observed. The mixed ANOVA yielded no significant main effects of hour, F(5, 60) = 2.12, p>.05, or group, F(1, 60) = 0.10, p>.05. While a global hour × group interaction was exhibited, F(5, 60) = 17.16, p<.001, post-hoc planned comparisons did not reveal any significant differences between paired and unpaired groups at any hour during the self-administration test (all F(1, 60) tests<0.87, p>.05; Figure 3).

## Discussion

### Goal-directed behavior engendered by the S^D^

The present experiment was designed to test whether abstinent responding triggered by a S^D^ is a stimulus-response habit, or a goal-directed action, by manipulating the value of cocaine [7]. Despite the absence of the S^D^ during cocaine-LiCl pairings, its ability to induce responding, as observed in the unpaired group, was absent in the explicitly paired group. Reduced S^D^-induced responding was likely due to explicit pairings of cocaine with LiCl because unpaired animals that received similar LiCl and cocaine exposure on separate days exhibited increased response rates upon S^D^ exposure. The observed S^D^-induced responding of unpaired animals was similar to animals with no LiCl exposure [22], demonstrating that LiCl did not affect the capacity of the S^D^ to engender responding. These data suggest that the value of the response outcome (cocaine) is not only evoked by the S^D^, but subject to manipulation following self-administration through cocaine devaluation.

In spite of three weeks of cocaine self-administration training, for paired animals, the S^D^ predicted the aversive properties of LiCl that followed cocaine exposure during devaluation. In contrast, since LiCl was administered 24 hours following cocaine exposure in unpaired rats, the S^D^ predicted an unaltered value of cocaine. Unpaired animals eventually reduced response rates to the level of paired animals during testing in extinction, indicating that the expectation of drug was not met when responses during S^D^ presentations did not produce cocaine infusions. The ability of the S^D^ to engender goal-directed responding may have been due to its unique, near 1.0 correlation with response contingent cocaine infusions and consequently, its unmatched prediction of reward in the current paradigm. Due to the close temporal proximity of the S^D^ with the infusion, cues that signal consumption of the outcome may remain sensitive to devaluation after overtraining, and by definition goal-directed as previously hypothesized [26].

### Habitual behavior engendered by non-S^D^ cues

Equally noteworthy was the lack of differences in response rates between paired and unpaired groups during the hour prior to S^D^ presentations in the cue-presentation test. This finding suggests that responding in the absence of the S^D^ and under abstinent conditions is habitual. Stimuli present during this time period were the self-administration chamber and lever. Unlike the S^D^, these stimuli were poor correlates of cocaine infusions. That is, high response∶reward ratios were observed during self-administration training, diluting the correlation of lever presses with cocaine infusions. Furthermore, during the self-administration phase of the experiment, animals lived in the self-administration chamber, spending 18 hours of each training day not self-administering. The weak correlation of these environmental cues with the cocaine infusion may be a factor in the development of habitual behavior [7], [27], [28] and the reason why the lever and self-administration chamber did not apparently evoke a representation of the devalued cocaine in the paired group and thereby influence response rates in the test hour prior to S^D^ presentations. Interestingly, the non-S^D^ cues were likely correlates of the tonically elevated levels of cocaine during training, similar to conditioned place preference. Since cocaine conditioned place preference is not blocked by LiCl [29], cues associated with the tonic levels of cocaine as well as those not strongly correlated with the outcome, may be particularly resistant to devaluation of the outcome (cocaine infusion in this case) and by definition, are effective producers of habitual behavior.

Studies have demonstrated that responding under the influence of cocaine is manifestly controlled by drug level [13], [15]. The present findings corroborate our earlier report in which animals' responding during self-administration did not discriminate the presence or absence of the S^D^ [22]. Given that our schedule of reinforcement precluded rats from attaining drug satiety, coupled with the animals' experience with long daily access and extensive training conditions, rats likely engaged in “compulsive” responding driven by and for cocaine [15], [23]. Despite the lack of evidence that responding was under stimulus control during self-administration, animals clearly learned a S^D-^cocaine association in training, expressed as the paired group's reduced responding upon S^D^ exposure during the abstinent cue-presentation test. Given that cocaine taking is eliminated by satiety and thus a goal-directed action [15], it is not surprising, although not previously demonstrated, that cue-induced responding following thirty days of abstinence is also goal-directed. However, the finding that responding during the absence of the S^D^ was habitual suggests that over cocaine self-administration training, habitual behavior was latently developing, but was not expressed until induced by environment-cocaine associations under abstinence conditions.

Given the evidence that responding prior to and following the first hour of testing was goal-directed, one might question whether responding in the first hour was habitual. If so, the habit must have formed during self-administration training, but as noted above, there is no evidence to support this. Therefore, it must be considered whether behavior mechanisms other than habitual responding contributed to the observed response rates during the first hour of testing. For example, it has been reported that rats respond at higher levels during extinction from cocaine self-administration when the testing chamber is not the home cage [30]. Our experimental design involved the removal of rats from the self-administration/home chamber during abstinence and response rates were equally high in both groups when returned. This may be especially important because our prior investigation on S^D^-induced responding yielded markedly diminished responding during the first hour of testing when animals were housed in the self-administration chamber over 30 days of forced abstinence prior to the cue-presentation test [22]. Regardless, the lack of difference between groups argues that responding in the first hour was not goal-directed. It is also possible that different methods of devaluation, such as single infusions of cocaine followed by single infusions of LiCl, could produce differential responding between groups during the first hour of testing. Finally, during training, different Pavlovian associations were likely formed between cocaine and the S^D^ from those formed between cocaine and the operant environment. The S^D^ may induce a more specific representation of the temporally proximal *infusion*, leaving the S^D^ more susceptible to evoking recollection of the devalued cocaine. In contrast, the operant environment and the lever, which were poor predictors of cocaine infusions, may have formed associations with *tonically* elevated levels of cocaine during self-administration training. If so, cues associated with tonically elevated levels of cocaine rather than the consumption of cocaine (the infusion) apparently do not evoke a representation of the value of cocaine. If these environmental cues had evoked a representation of cocaine's value, decreased responding would have been observed in the paired group during the first hour of abstinent testing relative to the unpaired group. Furthermore, this suggests that during devaluation training, paired animals learned that the infusion, rather than tonically elevated levels of cocaine, produces predicted sickness.

### Differential responding to cues

Since responding during the absence of the S^D^ was habitual while responding during the presence of the S^D^ was goal-directed, abstinent responding may be driven by either of two behavioral mechanisms. Relapse is considered a process rather than a single event [8]. Throughout the process of relapse, an abstinent addicted cocaine user is likely to encounter a multitude of cues ranging from ambiguously to perfectly correlated with cocaine consumption. Depending on the strength of each cue's correlation with cocaine infusions, a spectrum of goal-directed behaviors and habitual behaviors may be engaged. However, since our results demonstrate that goal-directed behavior is able to terminate habitual behavior (i.e., hour 2 compared to hour 1), the ultimate determinant of relapse may be a goal-directed action. Moreover, habitual, or “absent minded” relapse [2] requires cocaine to be immediately accessible, whereas goal-directed relapse employs volition, necessary to manage the logistics (Where and how to get drug and paraphernalia?) and challenges (Take health, monetary, and punitive risks?) inherent in the process. Nevertheless, our results suggest that both mechanisms participate in the process of relapse.

### Goal-directed cocaine seeking

The notion that drug-seeking behavior is a goal-directed action is bolstered by behavioral economic analyses of animal self-administration behavior in which changes in drug choice are explicitly tied to changes in response cost [31], frequency of reward [32], dose per infusion [33], delay to reinforcement [34], infusion duration [35], feeding schedule [31], [36], probability of reinforcement [37], availability of alternative reinforcers [38], [39], and satiety [15].

In cocaine addicted individuals, choice paradigms pitting drug versus alternative rewards have revealed goal-directed actions to obtain drugs. The choice to self-administer cocaine over receipt of monetary reward depends on the dose the cocaine user will receive if he or she participates in the study [40]–[45]. In other words, addicted individuals do not work or “pay” for cocaine when the perceived expected value of the drug is reduced, implying a goal-directed process. Indirectly, investigations of self-administration behavior in cocaine addicted individuals have also revealed goal-directed self-reports. Specifically, cocaine addicted individuals self-report high ratings of “I want cocaine” while intravenously binging [46], [47] or in response to cocaine-associated cues [48], [49]. In some reports, cravings specifically for cocaine have been rated higher than nonspecific self-reports such as “rush”, “high”, or “excited” [50]–[53], but not in all cases [54]. It is interesting that when a cocaine-addicted individual is currently under the influence of cocaine by drug priming or self-administration, the choice to self-administer cocaine over monetary reward is nearly always cocaine [41], [42]. The lack of differences between paired and unpaired groups during the cocaine self-administration test may reflect that cocaine itself is a stimulus that can engender responding. Upon earning their first infusion of cocaine, animals may have entered into a “compulsive” state of responding [15], given that 1) the “priming threshold” that initiates responding for cocaine is less than one infusion of earned cocaine at the present dose, 2) responding does not cease until drug level reaches the “satiety threshold”, and 3) our schedule of reinforcement precluded rats from attaining drug satiety. Furthermore, given that 1) discriminative responding to the S^D^ tone is masked during self-administration but can be revealed during abstinent testing [22] and 2) the expression of habitual behavior is also masked during self-administration [15] but was revealed during abstinent testing in the present experiment, cocaine's presence during the self-administration test may have additionally masked the previously learned association between the cocaine infusion and sickness. Thus, the cocaine self-administration test, rather than indicating that LiCl sickness failed to devalue cocaine in the paired group, may produce results that are not akin to similar ‘reacquisition’ studies with natural rewards [7], [26].

### Alternative explanations

During the devaluation phase of the experiment, prior to infusions of LiCl, the paired animals received infusions of cocaine whereas the unpaired group did not receive identical infusions of saline. Thus, it is possible that nonspecific sensory properties of the infusion (i.e. increased venous pressure following pump activation) may have been associated with LiCl-induced sickness in the paired, but not unpaired group. However, this is unlikely to produce decreased response rates during the cue-presentation test for several reasons. First, except during self-administration and devaluation training, animals received saline infusions every fifteen minutes for over two months. Prior to devaluation training, over 2400 infusions of saline were administered per rat. The large number of exposures to these infusions, during which there were no consequences to the nonspecific sensory aspects of the infusions, would likely undermine any possible infusion-LiCl-induced sickness associations during devaluation, which were far fewer in number. Second, both the paired and unpaired animals received intravenous LiCl administration during devaluation training. If LiCl-induced sickness was to become associated with aspects of the LiCl infusion, it is likely to occur immediately preceding pump pressure. Yet since both groups received the same intravenous route of LiCl administration, both groups would have equally associated the nonspecific effects of the infusion pump with LiCl-induced sickness. Instead, responding differed between groups during S^D^ exposure. Third, if the paired animals did make associations of the nonspecific sensory properties of the infusion with LiCl-induced sickness, these would likely have been extinguished following the completion of devaluation training and thirty days of abstinence during which over 3800 infusions of saline were administered per rat.

One might also consider the possibility that a S^D^ paradigm might not be sufficient to produce habitual behavior, as goal-directed cigarette-seeking behavior is repeatedly observed in addicted smokers in response to S^D^ cues [55]–[61]. However, research has shown that resistance to outcome devaluation (e.g. habitual responding) can develop for oral sucrose or food self-administration using similar discrete noncontingent S^D^ paradigms [62], [63]. Moreover, the present paradigm provided stimuli in addition (i.e. lever, operant chamber) to the S^D^ that proved sufficient to produce habitual behavior.

The observed goal-directed responding stands in contrast with experimenter-administered psychostimulant experiments leading to habit formation in responding for sucrose [9]–[11]. Since subjects in the current experiment self-administered nearly 600 mg/kg of cocaine over three weeks and the aforementioned study utilizing cocaine involved approximately 400 mg/kg of experimenter-administered cocaine [9], the finding that S^D^-induced responding did not become habitual cannot be attributed to insufficient cumulative drug exposure. Instead, one reason that cocaine-seeking remained goal-directed while food-seeking behavior became habitual after drug exposure may be due to fundamental differences between drug-seeking and food-seeking behavior. On the other hand, that habitual responding occurred in the hour prior to S^D^ presentations and food-seeking behavior can become habitual under similar experimental circumstances following amphetamine-exposure [10], [11] suggest that the expression of habitual behavior is related to presentation of cues that do not strongly correlate with consumption of cocaine or food.

### Neural mechanisms of relapse

The dorsolateral striatum has been linked with acquiring habitual responding for food reward [24] and has thus been hypothesized to be involved in “habitual drug-seeking” [5]. Macey and colleagues [64] observed decreased glucose metabolism in the dorsolateral striatum after 60 hours of cocaine self-administration (2 hour daily sessions over 30 days). Similarly, a decrease was observed in dorsolateral striatum single neuron firing rates during instrumental movements over 28 hours of water self-administration (2 hour daily sessions over 14 days; [65]). In the water-seeking experiment, animals acquired a habit, as evidenced by maintained operant movements despite prior satiation with water. In the cocaine-seeking experiment [64], although habit formation was not tested, neural activity of dorsolateral striatum neurons could be interpreted as a correlate of habit formation.

A current theory of the neural basis of addiction posits an increasing role of the dorsolateral striatum and a decreasing role of the ventromedial striatum concomitant with a shift from goal-directed to “habitual drug-seeking” [5]. However, ventromedial striatal neurons (especially of the nucleus accumbens (NAcc) core) continue to exhibit robust changes in firing rates during cocaine self-administration or drug-seeking not under the influence of cocaine after many weeks of self administration and abstinence [66], [67]. In contrast, in a variety of paradigms, the vast majority of dorsolateral striatum neurons exhibit decreased neuronal activity [64], [65], [68] or lose their unconditional movement firing characteristics with overtraining [69], [70]. While the dorsolateral striatum is likely to be involved in some aspects of cocaine-seeking behavior [71], involvement of the dorsolateral striatum in cocaine-seeking behavior does not itself constitute evidence that cocaine-seeking behavior is habitual (as argued in [5]). The continued involvement of the NAcc [22], [66], [67], [72], the involvement of other brain regions known to encode the “value” of learned cocaine-associated stimuli [73]–[83], and the present findings suggest that a value-based neural circuitry may be a critical component in mediating S^D^-induced responding. However, one might speculate that nonvalue based brain regions linked with habitual responding, such as the dorsolateral striatum, may be particularly active during the hour of testing prior to S^D^ presentations. Nevertheless, drug-seeking behaviors, which are by definition goal-directed and linked with value-based circuitries, and habitual behaviors, which are by definition not goal-directed and linked with nonvalue-based circuitries, are both likely contributors to the process of relapse.

### LiCl-based aversion therapies

Although the present results may indirectly support testing the utility of LiCl aversion therapy in reducing cue-induced relapse in cocaine addicted individuals, this was not our intention. While LiCl is known to block the stereotypical behaviors induced by cocaine [84], low dose 24 hour continuous infusion of LiCl does not block self-administration of cocaine [85]. Furthermore, although LiCl produces robust conditioned place aversion [25], LiCl administration does not block conditioned place preference induced by cocaine [29]. While certain types of aversion therapy have been shown to completely eliminate cocaine cravings in the laboratory [86] it is not known if aversion therapy has lasting effects that decrease relapse outside the laboratory [87]. Indeed, craving can be driven by internal cues such as dysphoria [88], [89], which is likely to be induced by aversion therapy. Furthermore, in the present study, pairing LiCl-induced sickness with cocaine eliminated S^D^-induced responding. but did not eliminate responding altogether. The attenuated level of responding was not decreased enough to prevent self-administration of cocaine on the second day of testing, a testament to the powerful influence of cocaine. Once under the influence of cocaine, addicted individuals nearly always choose cocaine over other reward choices [41], [42] and animals do not cease responding until “satiated” [15].

### Conclusion

Habit learning can be pathological, but as a normal process has been described as adaptive [90], allowing for the cognitive elevation of a primary task via subordination of a more common, well-learned behavior. Therefore, it is not unexpected that rats, upon return to the operant environment (and cues weakly correlated with cocaine infusion), should readily return to the task of lever pressing. What is of particular interest is how the S^D^ 1) immediately interrupted habitual responding which preceded its onset and 2) singularly manifested differences in responding consistent with the value of cocaine. These findings support the claim that relapse is a complex behavioral process involving habitual and goal-directed behaviors that are differentially influenced by cues that vary in their correlation with the cocaine infusion. The relative contribution of habit learning versus goal-directed processing in driving relapse remains to be determined and might ultimately guide treatment strategies. Therapies aimed at altering habitual behavior patterns may limit encountering cues even weakly associated with cocaine. Alternatively, the development of therapeutic approaches may be better informed by evidence that the influence of cues signaling a strong relationship with cocaine infusion availability engages goal-directed actions rather than stimulus-response (i.e. habitual) behaviors.

## Materials and Methods

### Ethics Statement

Protocols were performed in compliance with the Guide for the Care and Use of Laboratory Animals (NIH, Publications 865–23) and were approved by the Institutional Animal Care and Use Committee, Rutgers University.

### Subjects and surgery

Male Long-Evans rats (n = 14; 325–335 g; Charles River, Wilmington, MA) were implanted with a catheter in the right jugular vein. All details of the surgical procedure and post-operative care have been described in detail elsewhere [91]. Following surgery, animals were randomly assigned to one of two groups: paired or unpaired. Animals were administered 200 µL of heparinized-saline infusions every fifteen minutes throughout the experiment, except during training and testing conditions.

### Procedure

During the self-administration phase of the experiment, before the beginning of each daily self-administration session, a nonretractable response lever was mounted on a side wall of a standard operant chamber in which the animal lived. Each lever press in the presence of an audible tone (3.5 kHz, 70 dB) produced an intravenous infusion of cocaine (0.355 mg/kg infusion), terminated the tone, and started an intertone interval (3–6 min). If lever pressing did not occur during a 2 min tone presentation period, the tone was terminated, and an intertone interval began. Each cocaine self-administration session lasted until 80 infusions were earned or 6 hours elapsed, whichever occurred first. Self-administration occurred seven days a week daily for three weeks. To facilitate acquisition of self-administration behavior, animals were shaped to lever press in the presence of the S^D^ for a 0.71 mg/kg dose on the first day of training. During the shaping session, the S^D^ was continuously sounded until responding occurred, at which time the S^D^ was terminated, cocaine was infused, and a thirty second time out period began. Responses during the time out were recorded but had no programmed consequences. Following time out, the continuous S^D^ was again initiated. After ten cocaine self-infusions and for the remainder of the self-administration phase, rats were trained under the 2 min S^D^ duration, 3–6 minute time out schedule of reinforcement. Rats were never drug primed. Animals were housed in the self-administration chamber during the self-administration phase of the experiment.

Following three weeks of daily self-administration, animals were transferred and housed in a wire mesh holding cage for the LiCl phase of the experiment. The overall experimental schematic for the LiCl phase is presented in Figure 1 . For the paired group, on day 1 of the three day cycle, each animal was noncontingently infused with cocaine for 1.5hours according to its self-administered pattern of drug intake on the last day of training. Cocaine infusions were immediately followed by infusions of LiCl (18 mg/kg/infusion). Cessation of LiCl administration occurred when cocaine-induced stereotypy as well as locomotion (operationally defined as alternating limb movements) ceased for at least one minute [25]. On days two and three of the three day cycle, paired animals were not administered LiCl or cocaine. For the unpaired group, on day 1 of the three day cycle, LiCl was administered until locomotion ceased for at least one minute. On day two of the three day cycle, unpaired animals were noncontingently infused with cocaine for 1.5 hours according to each animal's self-administered pattern of drug intake on the last day of training. On day three of the three day cycle, unpaired animals were not administered cocaine or LiCl. For paired subjects, four repetitions of the aforementioned cycle occurred. In comparison to unpaired controls, cocaine-infused subjects required more daily LiCl injections to cease locomotion. To equate LiCl exposure for both groups, in addition to four repetitions of the aforementioned cycle occur, unpaired subjects received 1–2 additional cycles of LiCl exposure, with no additional cocaine exposure during the additional cycles.

Note that our outcome devaluation procedure selectively pairs the outcome, cocaine, with LiCl-induced illness in the paired group. Other methods such as LiCl delivery following cocaine self-administration (i.e. punishment) pair LiCl-induced illness with cocaine, instrumental responding, the self-administration chamber, and the prior presentations of the discriminative stimuli, which would generate ambiguous interpretations of testing data. The present outcome devaluation method allows for testing whether 1) stimuli in the environment, which were never paired with LiCl-induced sickness, or 2) the outcome, cocaine, which was paired with LiCl-induced sickness in the paired group, controls responding during abstinent testing. Also, the cocaine self-administration test following cocaine devaluation does not definitively test whether cocaine self-administration is habitual or goal-directed because cocaine is a stimulus which can engender responding on its own [15].

Animals remained in the holding cage for 30 additional days after the last cocaine exposure in the LiCl phase. Subsequently, animals were returned to the self-administration chamber 18 to 72 hours before the test of cue-induced responding (details of test in [22]). On day 1 of testing (cue presentation test), the lever was installed and animals were free to lever press without programmed consequence. The S^D^ tone was not presented to the animal during the first hour of testing. During the remaining five hours of the test, the S^D^ tone was presented for 30 seconds every 3–6 minutes. Responses emitted during tone presentations terminated the tone and infused saline (3.755 s), whereas responses emitted while the tone was off had no programmed consequence. On day 2 of testing, animals were allowed to self-administer cocaine with all parameters identical to training.

### Statistical analysis

All outcome variables in the study, e.g., responses/minute, self-administered mg/kg/day, etc., were analyzed as a function of a set of categorical fixed effect independent variables, e.g., group, week, etc., and their interactions using mixed ANOVAs. SAS PROC GLIMMIX (SAS Institute Inc., 2005) was used to run all analyses. All outcome variables were highly skewed and therefore theorized to be gamma distributed rather than normally distributed. Thus, for all outcome variables a gamma distribution with a log link was specified for the outcome variable in the mixed ANOVA. Outcome variables were collected on multiple occasions from each subject, and thus, subject was specified as a random effects variable for those variables. The final solution for the mixed ANOVA model was estimated using maximum pseudo-likelihood marginal expansion. The degrees of freedom in the model were computed using the containment method. Because the data were not normally distributed, the standard errors were computed using the first order residual empirical estimator, also known as the sandwich estimator. All other default settings in PROC GLIMMIX were maintained. Post-hoc simple effects were computed for any overall significant interactions. Alpha criterion for all tests was 0.05.
